# Editorial: Healthcare management: challenges for improving performance and quality of patient services

**DOI:** 10.3389/fpsyg.2023.1272083

**Published:** 2023-09-20

**Authors:** Juan Herrera, Carlos De Las Heras-Rosas, Pedro Mota Veiga

**Affiliations:** ^1^Department of Economics and Business Administration, Faculty of Economics and Business Administration, University of Malaga, Málaga, Spain; ^2^NECE - Research Center for Business Sciences, University of Beira Interior, Covilhã, Portugal

**Keywords:** healthcare management, job satisfaction, organizational commitment, performance, quality of patient care

In healthcare, institutions face constant challenges due to technological advances and increasing societal expectations, particularly in times of pandemic. Personnel management becomes essential in these critical circumstances. Within this context, human capital management challenges are diverse. The lack of qualified staff, the coordination of specialized multidisciplinary teams, and the management of factors such as stress, burnout, job dissatisfaction, and high turnover present themselves as key obstacles to meeting the high standards of performance and quality of patient care that society demands.

This Research Topic of Frontiers in Psychology includes seven articles. These publications were carried out using qualitative and quantitative techniques and systematic reviews. As units of analysis, the published articles have used nursing staff (4 doc.), first-line managers (1 doc.), general practitioners (1 doc.), and emergency staff (1 doc.).

In healthcare institutions, occupational burnout stands out as a crucial challenge. Teymoori et al. focus on this issue by investigating the elements linked to occupational burnout in operating room nurses. Their analysis reveals various factors of burnout, including organizational, interpersonal, occupational, and personal aspects. They have found that participants reported a lack of support and experienced inappropriate behavior from surgeons and other colleagues. To address these problems, the authors suggest, among other proposals, the design of prevention strategies, the incorporation of senior managers committed to improving working conditions, and strengthening interpersonal and stress management skills. Along these lines, Verhoef and Blomme analyse the generic and specific determinants of burnout syndrome among general practitioners. From their results, it emerges that there is a connection between gender and burnout, mediated by work pressure and social support from partners. In addition, other stressors, such as workload and time management, and protective factors, such as clinical interest, are identified. On the other hand, they highlight that continuity of care is eroded due to increased workload as a result of patient expectations and bureaucracy. Finally, they add that there is stress from feeling morally bound to be good doctors and that this has implications for the mental health of GPs. They point out that there are few studies that analyse the problems of this group in depth.

Other research in this Research Topic highlighted the context of the COVID-19 pandemic. In this regard, Jin investigates the job satisfaction status of psychiatric nurses, their job burnout, and the moderating effect of family support during the pandemic. Her results suggest that nurses were dissatisfied with their job status. Stressors such as low pay, lack of promotion opportunities, and attention to suggestions are identified. In addition, the findings reveal that some of them experienced a considerable level of job burnout. The author highlights that the main contribution to the study was the paradoxical finding that family support could have a counterproductive effect when nurses experience a decrease in job satisfaction. Among the author's proposals are establishing a “hospital-family alliance” to adapt policies according to needs, optimizing workflow, hiring more staff, or providing psychological support and organizational adjustment according to the level of burnout. On the other hand, Kukulskienė et al. in their research analyse pandemic-related subjective difficulties and challenges in work organization. The aim of the research was to understand how health professionals experienced daily life, wellbeing, work activities, and management during the multiple changes in organizational and individual practice experienced in this unexpected and unique situation. The results shed light on the fact that during the first wave of the COVID-19 pandemic, healthcare workers faced overwhelming changes in all areas of life, including job uncertainty and lack of clarity in tasks and roles. These changes generated an intense emotional response, reflecting the tension between adaptation to new roles and loss of control. The threats of the pandemic highlighted the importance of active leadership and a supportive organization for staff.

Another point of interest focuses on organizational performance. Liu B. et al. highlight the relationship between patient expression of gratitude and the innovative performance of nurses. Their results confirm the existence of such a relationship, considering that patients' grateful emotions would improve the meaning of nurses' work. To this, they add that these relationships are stronger the more supportive the supervisor is. Among their proposals, the authors point out that increasing mutual understanding between patients and professionals by sharing thank-you letters is positive. Hospitals should foster inclusive work environments to promote “self-connection,” raise nurses' awareness of the importance of their work, and organize team-building activities. Moreover, supervisors should provide instrumental support to nurses by clarifying tasks and showing confidence in their creative ideas. In this line, on performance, Wolffgramm et al. in their work try to identify the differences in role expectations between first-line managers (FLMs) with their middle managers and HR professionals related to organizational performance. Their results suggest that FLMs have a crucial mediating role in the relationship between HR practices and organizational performance. Due to their intermediate position, they have to balance management and HR roles, which can cause stress. Communication and alignment between middle managers and HR professionals are essential to ensure the relationship between HRM and organizational performance. Among their proposals, they suggest that organizations should actively intervene to mitigate differences in role expectations between different actors by addressing role ambiguity and fostering clear and effective communication in the context of HRM.

Finally, in relation to turnover, Liu Y. et al. try to determine the factors associated with nurses' intention to leave the organization. Their results suggest that a high percentage of nurses have expressed their intention to leave their current jobs. Factors such as education, salary level, marital status, job type, job satisfaction, and sense of belonging to the hospital are shown to influence the intention to leave. The authors highlight the need to focus on professional development, job satisfaction, conflict management, and the creation of a healthy work environment to retain nurses.

In summary, this set of seven articles increases knowledge about healthcare management and its challenges to improve organizational performance and the inescapable goal of improving patient quality of life.

## Author contributions

JH: Conceptualization, Investigation, Project administration, Supervision, Writing—original draft, Writing—review and editing. CD: Conceptualization, Investigation, Supervision, Writing—original draft, Writing—review and editing. PV: Conceptualization, Investigation, Supervision, Writing—original draft, Writing—review and editing.

